# Leaderboard Design Principles to Enhance Learning and Motivation in a Gamified Educational Environment: Development Study

**DOI:** 10.2196/14746

**Published:** 2021-04-20

**Authors:** Sungjin Park, Sangkyun Kim

**Affiliations:** 1 Department of Industrial Engineering Kangwon National University Chuncheon Republic of Korea

**Keywords:** leaderboard design, gamification, learning motivation, affordance

## Abstract

**Background:**

Gamification in education enhances learners’ motivation, problem-solving abilities, decision-making abilities, and social skills such as communication. Numerous ongoing studies are examining the application of gamification design methodology and game mechanics to a learning environment. Leaderboards are a type of game mechanic that assist learners in goal setting and unleash the motivation for learning.

**Objective:**

The aim of this study was to develop leaderboard design principles to assist learners in efficient goal setting, improve learning motivation, and promote learning in gamified learning environments.

**Methods:**

This study implemented 2 different strategies. First, we analyzed previous research on leaderboards that focus on educational efficacy and influence on social interactions. Second, we collected and analyzed data related to cases of leaderboards being used in educational and sport environments.

**Results:**

This study determined 4 leaderboard design objectives from previous studies. Based on these objectives, we developed 3 leaderboard design principles. First, macro leaderboards and micro leaderboards should be designed and used together. Second, all the elements used to measure learners’ achievements in an educational environment should be incorporated into the micro leaderboard. Third, leaderboards should be designed and considered for application in contexts other than learning environments. This study further analyzes best practices considering the 3 leaderboard design principles.

**Conclusions:**

This study contributes toward resolving problems associated with leaderboard design for the application of gamification in educational environments. Based upon our results, we strongly suggest that when teachers consider applying gamification in classrooms, the leaderboard design principles suggested in this research should be incorporated.

## Introduction

Recent advances in technology have caused our methods of communication and our lifestyles to evolve at an unprecedented rate. However, this has not necessarily been the case in the field of education. Although there has been some technology advancement in the classroom, such as the introduction of digital devices to replace books, knowledge is mostly delivered to learners in one direction by the teacher. Currently, researchers are actively studying diverse educational methods such as problem-based learning and learner-centered educational environments [1]. Gamification, which means using gaming elements, structures, and principles in educational innovation, is attracting the attention of teachers and instructors. Gamification uses game mechanics such as points, badges, levels, or avatars to motivate participants by providing flow and fun experiences while promoting social interaction among participants [2,3].

**Figure 1:**
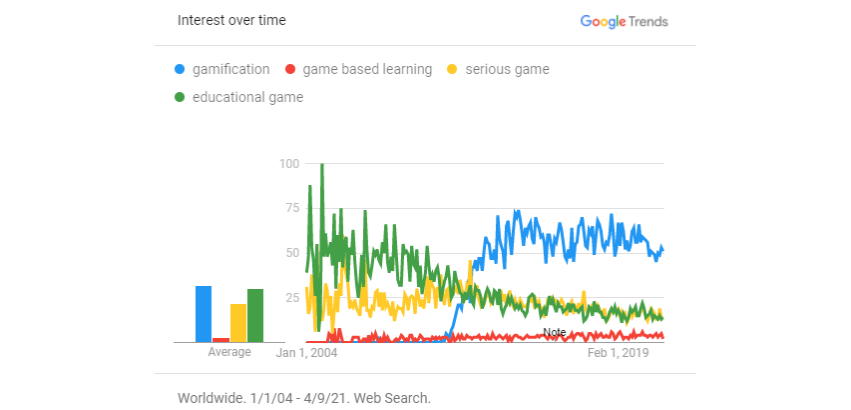
Trends of gamification, game-based learning, educational games, and serious games by using the Google Trends search.

The application of gamification in the learning context is called “gamification in education” [4]. Al-Azawi et al [5] conducted a comparative analysis of gamification, game-based learning, and educational games, which are mechanics that represent educational innovations based on game thinking. They found that gamification is easier to achieve and more affordable than other methods and that it encourages learners to undertake new challenges without fear of failure. Considering the research of Al-Azawi et al, this study confirmed the trends of gamification, game-based learning, educational games, and serious games by using a Google Trends search (Figure 1). The results showed that gamification has been receiving worldwide attention and popularity since 2012. Park and Kim [6] collected 754 cases of gamification from pre-2010 to 2017, 270 of which were related to education.

Gamification in education is beneficial to learners in several ways. Majuri et al [7] collected 807 studies related to gamification in education from June 2015 and performed an empirical study on 128 relevant studies. Their findings revealed that game mechanics unleash and improve affordance, immersion, and the social behavior of learners. Psychological benefits include improvements in cognitive and emotional ability, stimulation of the desire to be challenged, increased social interaction, projection of psychological states and traits, and the enhancement of personal traits. Behavioral outcomes include improved learning performance, engagement, and physical and social interactions. Kim et al [4] suggested the Lean Canvas-based 4F Process for more efficient gamification design. The Lean Canvas-based 4F Process is a methodology that analyzes the objects of gamification, player type, fun preferences, and game genre preferences to help users set game mechanics and develop prototypes. Meanwhile, Mora et al [8] analyzed 18 studies on gamification design methodology for the period 2011 to 2015 and identified 5 gamification design elements. These elements are as follows:

1. Economic: objectives, risk, return on investment, and stakeholders
2. Logic: loop, end game/epic win, on-boarding, rules
3. Measurement: metrics and analytics
4. Psychology: fun, motivation, socialness, desired behaviors, and ethics
5. Interaction: narrative, user interface/user experience, and technology

Octalysis [9] is a published gamification design framework for the development of gamified learning content that is based on human behavioral characteristics, cognitive structure, and the Gamified Environment and Learning Design model [10]. Game mechanics design is one of the most important elements shared by the published gamification design methodologies. Game mechanics represent the physical and environmental elements connecting users and games [11]. Through game mechanics, affordance is achieved and values nested in the content are transmitted to players [12].

Leaderboards, among other game mechanics, assist users with goal setting, boost competition, and provide feedback. Dicheva et al [13] conducted systematic mapping of 34 studies related to gamification in education published between 2011 and 2014. Their results showed that leaderboards were the most frequently used method—after badges—in the 34 studies. Goal setting is extremely important in educational environments [4]. A leaderboard is a device that guides learners to set specific goals and that represents the outcome in a visible way [7]. However, detailed leaderboard design methods are not addressed in existing gamification design methodology. Kim et al [4] state that a leaderboard should be designed at micro and macro levels but did not mention the structural arrangement of the elements. Despite all the existing research on the effects of leaderboards, their organization has been rarely addressed. This study, therefore, develops a set of leaderboard design principles based on a literature review and the best case analysis of leaderboards in educational gamification.

## Methods

### Learners’ Self-feedback and Goal-Setting Via the Leaderboard

Leaderboards, as game mechanics, induce social behaviors and encourage interaction among participants through competition and cooperation [14]. Learners are self-motivated to check their positions on the leaderboard and stimulated to inform others of their accomplishments [15]. According to O’Donovan et al [16], leaderboards influence learners’ motivation more than other game mechanics such as progress bars, end prizes, and awarded badges. However, leaderboards cannot be used alone; they provide information for learners in conjunction with other game mechanics. Teachers assess the performance of the learners and award points, levels, and badges accordingly. Additionally, teachers display who scored how many points and badges and who is at which level on the leaderboard. Learners can see their ranking on the leaderboard, compare themselves with other learners, and receive feedback on areas where they need to improve [17]. Gamification helps learners to immerse themselves in learning with enthusiasm and persistence [18]. As learners enter a state of flow in their learning, they set specific goals based on their level on the leaderboard [19]. McGonigal [20] presents goal setting, voluntary participation, rules, and feedback as game characteristics. These 4 characteristics can be applied to gamification in education. Game players gain a sense of purpose by setting goals. In gamified learning environments, learners receive feedback on their activities through the leaderboard and are rewarded for their achievements. Based on this dynamic, it is possible to induce voluntary participation and observance of classroom rules to leverage the utility of gamification in education [4]. However, a drawback of leaderboards is that they may dampen participant motivation or desire for learning because the mechanics are not applied to learners who are high on the leaderboard. Additionally, those in the lower ranks tend not to respond to the leaderboard and are likely to feel inadequate when comparing their achievements with those of the high-ranked learners [21]. Those in the upper ranks, those with higher than average ranks, or those who feel they are not significantly different from the higher-ranked learners may feel satisfied with their position or motivated to improve through upward counterfactual thinking [22]. Counterfactual thinking [23] is the act of considering past events that did not happen. Individuals with a positive mindset tend to employ upward counterfactual thinking. For example, a learner ranked seventh on the leaderboard may consider the learner ranked third and think, “If I had worked a little harder, I might have ranked third.” Conversely, when people have negative experiences, they tend to employ downward counterfactual thinking. For example, a learner ranked 20th on the leaderboard might think, “I dropped this time. I will probably drop further [24].” If this negative experience continues, the individual may lose the confidence and motivation for learning [25]. Based on the literature review, this study suggests the following objectives that should be considered in the design of leaderboards:

Objective 1: Minimize relative deprivation.

Objective 2: Minimize learners’ experiences of failure to minimize downward counterfactual thinking.

Objective 3: Maximize learners’ experience of success to induce upward counterfactual thinking.

### Structure of Leaderboards in a Gamified Learning Environment

**Figure 2:**
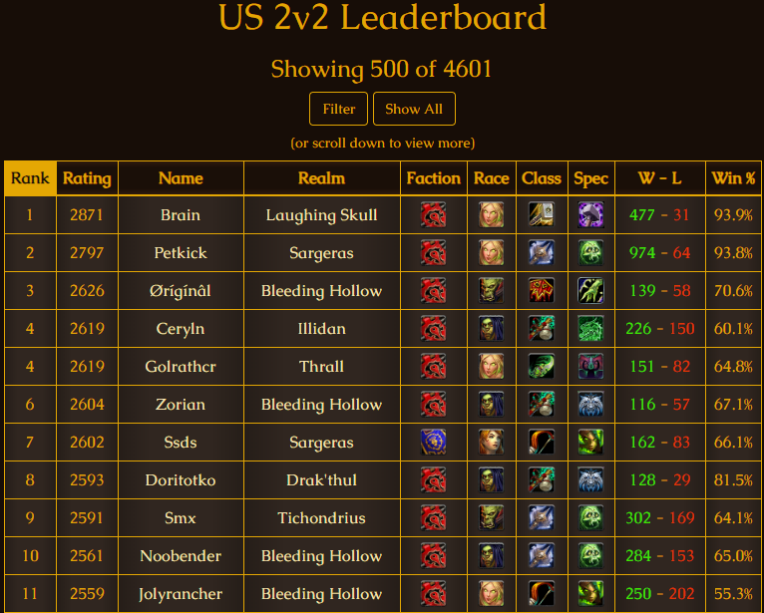
World of Warcraft leaderboard.

**Figure 3:**
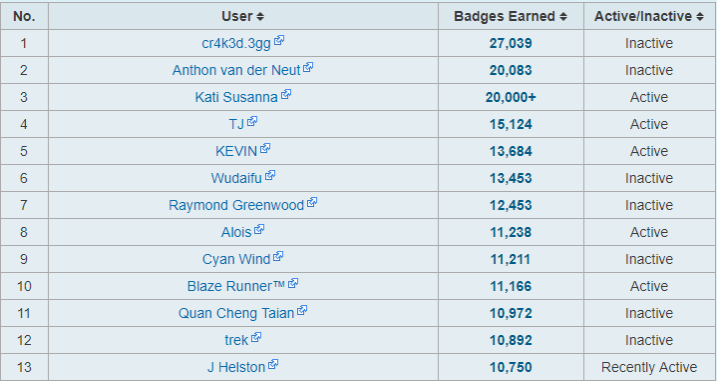
Badge count leaderboard of Khan Academy.

Leaderboards can be divided into 2 types: macro leaderboards, which are associated with overall content, and micro leaderboards, which are associated with a subsection of content [4]. Figure 2 displays the player versus player ranking in the World of Warcraft. Figure 3 is a badge count leaderboard for Khan Academy—a gamified learning platform for mathematical education. This figure shows the number of badges granted to the learners who satisfied the criteria specified by the Khan Academy. According to the standards suggested by Kim et al [4], Figure 2 is a macro leaderboard while Figure 3 is a micro leaderboard. Although these leaderboards serve different purposes, the structural characteristics in these leaderboards are similar. The player profiles are provided. The leaderboards in games offer information, including ranking, ID or nickname, organization, occupation in the game, character information, and the player’s win rate. The information includes rank, grade, experience, and earned badges. Game players meet and compete in the game and the information is reflected on the leaderboard. The same is true of the gamified learning environment. After conducting learning activities in the classroom, teachers assess the results and update them on the leaderboard. Based on the information registered on the leaderboard, learners maintain motivation for learning, compete with other learners, and set specific goals for themselves. Therefore, it is important that the leaderboard provides specific information. At the same time, the leaderboard should function smoothly to maintain the user-friendly and fun experience of the gamified learning content or environment.

Butler [26] examined the relationship between leaderboards and the fun experienced by players and 10 key findings were obtained. Those relevant to this research are as follows:

1. Players who have experienced competition play games at least once more than those who have not.
2. The level of fun experienced by players is not related to the number of plays.
3. Creating games that are too easy may downgrade the fun, depending on the player’s opinion.
4. High scores are not necessarily related to fun.
5. When players consider a game too easy, it is difficult to change their opinion; however, alternative definitions can be considered when it is deemed too difficult.

Appropriate competition induces a state of flow. In learning environments, macro leaderboards are used frequently. However, macro leaderboards can only provide numeric data totaling the performance of participants and can therefore only induce fragmented competition. Thus, many presume that frequent competitions between participants cannot be induced by design. Additionally, participants in upper ranks may be exalted by high scores, while those in middle and lower ranks cannot even entertain the thought of competing with those in higher ranks because of the gap in achievement. Therefore, leaderboards that reflect standards or elements that encourage competition in other areas are just as important as leaderboards that promote high scores. Considering the necessity of the following structural conditions in designing leaderboards based on our literature review, this study suggests the following structural purpose of leaderboard design.

Objective 4: Design leaderboards with measurable learning performance to induce learners to obtain high scores and compete with one another.

## Results

### Leaderboard Design Principles for Effective Gamified Learning

**Figure 4:**
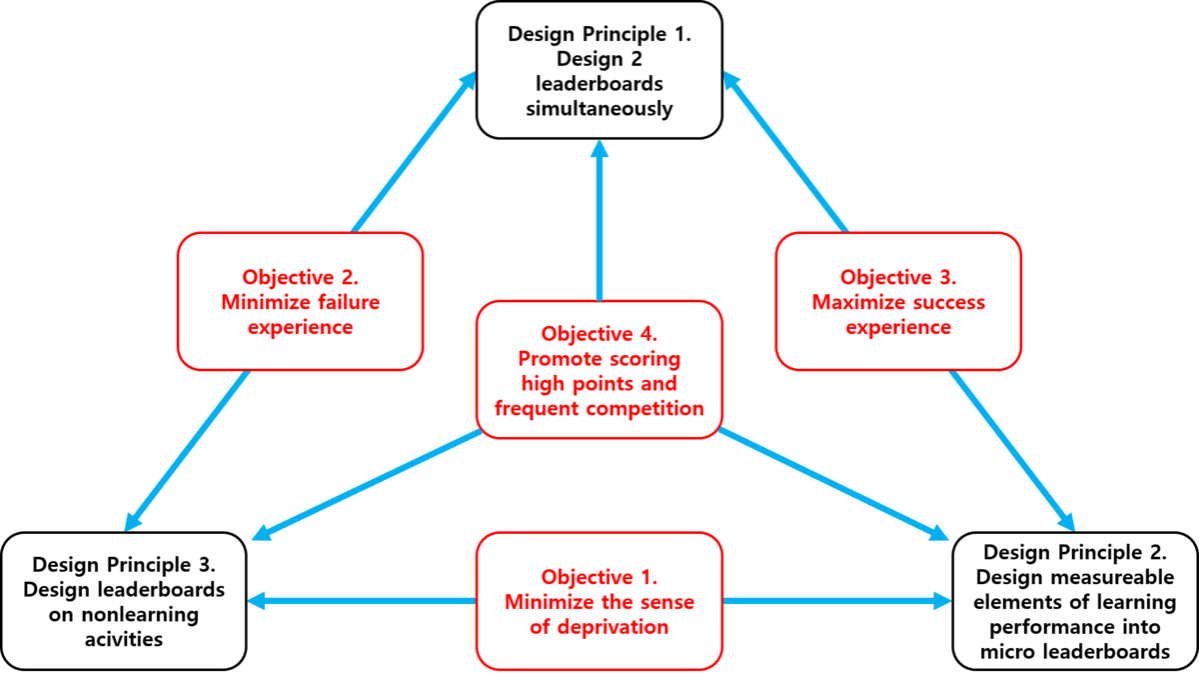
Suggested structure for leaderboard design.

To achieve the 4 objectives identified from the analysis of prior research, this study suggests 3 design principles, as summarized in Figure 4.

First, both macro leaderboards and micro leaderboards should be designed. If only macro leaderboards are used, participants in the mid and upper rankings can maximize their experience of success. Conversely, those who belong to the lower rankings on a macro leaderboard experience a sense of failure. If they are continuously recorded in the lower rankings on a macro leaderboard, their failure experiences accumulate and negative perceptions and experiences of learning are maximized [27]. To address this problem, learners require feedback to assure them that they are learning properly in some areas. Micro leaderboards fill this need by providing more detailed feedback than macro boards. Using micro leaderboards makes it easier to encourage the scoring of high points and frequent competition and to maximize success while minimizing failure.

Second, all the elements used to measure learners’ achievements in an educational environment should be incorporated into the micro leaderboard, as described in design principle 1. This allows participants to take part in learning activities in areas where they have confidence. Thus, learners realize an extended range of movement through leaderboard diversification and are consequently stimulated to achieve more in their learning [28]. Additionally, when the number of leaderboards increases, it is more likely that a learner will be listed in the upper ranks, resulting in increased motivation. The purpose of this design is to maximize success and minimize the sense of deprivation.

Third, leaderboards that reflect activities unrelated to learning performance should be designed to promote gamified learning. The goal of leaderboards is to encourage goal setting and engagement in learning activities. However, learners who are not interested in learning activities are not willing to engage in activities, no matter how much fun they are. This problem can be addressed by creating leaderboards for activities other than learning. For example, leaderboards for classroom tasks such as cleaning, counseling friends, library visits, and books borrowed from the library should be provided. Through leaderboards, participants can set a new and specific set of goals [29] and achieve success by moving up in the leaderboard ranks. In addition, activities other than learning can encourage frequent competition and easier point scoring. These features promote learning engagement, maximize success, and minimize a sense of deprivation.

#### Design Principle 1: Leaderboards Should be Designed on Both Macro and Micro Levels

Leaderboards frequently used in educational environments are macro leaderboards. In gamified learning environments, learner performance is incorporated into points or experience points that are shown on the leaderboard. Macro learning behavior is learning a theory named A in the gamified environment. The process of learning theory A is composed of microlevel learning behaviors such as A-1, A-2, and A-3 [30]. When using a macro leaderboard, it is possible to provide evaluation and feedback on macro learning behaviors but not on micro learning behaviors. It is therefore difficult to discern what activity a learner excels at from a macro leaderboard. This is because a macro leaderboard provides feedback on overall learning performance but does not give feedback on each learning activity. Kasworm and Blowers [31] suggested considering a variety of personal and environmental factors, as learners’ performance is the result of a combination of both factors. It is not easy for teachers to understand personal factors; however, environmental factors can be addressed by designers. If environmental factors are addressed, they can influence participants’ learning activities. Therefore, this study concludes that macro and micro leaderboards should be operated simultaneously. This provides learners with more experiences of success. Conversely, as the number of trials increases, the number of failures is expected to rise. Negative influences from increased failure should be controlled by gamification. If teachers can encourage learning activities through gamification [5], they can ensure more success than failure. Enhanced cooperation and more frequent competition between participants will create synergy for the achievement of individual goals [26,32].

#### Design Principle 2: Integrate Each Measurable Element in the Gamified Learning Environment With a Micro Leaderboard

In the same vein as Kasworm and Blowers [31], teachers who design gamified learning environments should integrate all elements into micro leaderboards. These elements include demographic factors. In learning environments, students are given grades based on their performance during a semester. Various evaluation items—from team activities to final exams—are designed to calculate grades. However, it is difficult for learners to obtain proper feedback because the items displayed on the leaderboard are only representative of activities or points that incorporate all activities. If a leaderboard is designed to incorporate each measurement applied in a gaming environment, learners will attempt to reach the upper ranking of that specific leaderboard. In the macro leaderboard, the greater the gap between the upper and lower ranks, the lower is the satisfaction with learning [33]. Meanwhile, learners’ confidence and learning activities are positively influenced by those in lower ranks relative to themselves [34]. As learners participate in more leaderboards where they compete with other learners in terms of performance, they gain confidence and are stimulated to try harder to earn high points. It is also important to provide multiple micro leaderboards to expose learners to objective evaluation and the achievements and competence of their peers. Using leaderboards facilitates the comparison of learning performance [35]. For example, if gamification is applied to a class during a semester and team activity scores, task scores, test scores, and badge acquisition status are elements applied to the leaderboard, other elements such as attendance points, the rankings of specific earned badges gained, the number of questions asked, the number of presentations, and other learning activities should be integrated into micro leaderboards. In the case of a web-based learning platform, all activities related to learning such as the time spent learning, the number of badges earned, or the number of assignments submitted (points) should be integrated with offline learning environment elements. Moreover, if the gender and age of the learner can be checked, leaderboards should be designed according to these factors. Actual web-based games provide a leaderboard based on most elements of the game such as server (region), occupation, and the gender of the game character.

#### Design Principle 3: Leaderboards Should Incorporate Activities Other Than Learning

The social characteristics of leaderboards have a positive influence on the learning effect through synergy with other game mechanics [35]. Leaderboards that focus on learning effects are effective for learners who rank highly; however, they can cause stress to newcomers or those who rank lower [27]. As a result, participants may have a negative perception of leaderboards. To encourage these disillusioned learners to focus on leaderboards again, a different approach is required. This requires a strategy to induce participants to engage in full-scale learning activities after eliminating the negative perceptions of leaderboards. This is done by introducing nonlearning activities. The leaderboard provides learners with their roles, responsibilities, and feedback on their status. [36]. The leaderboard causes participants to consider the influence of the leaderboard on other participants for activities other than learning. Nonlearning activities can weaken the sense of inadequacy caused by the leaderboard. Additionally, it will become easier for the participant to rank higher on the leaderboard for nonlearning activities than it is for learning-related leaderboards. Participants can maximize their success and find a state of flow through the process. At the same time, the needs of the participants in the mid and lower ranks will be met to minimize the sense of deprivation. Zhao and Tang [37] conducted an analysis of gamification cases currently in service based on the 8 core drives of octalysis [9]. They showed that one of the sources of motivational affordance was scarcity. Scarcity is explained as the core driver of wanting something simply because it is unattainable. The less scarce the user perceives an object or activity, the more patient the user becomes. Lower-ranked learners may have already given up learning. However, if they experience a higher ranking on nonlearning leaderboards, they may develop a different perspective on learning leaderboards. Therefore, designing micro leaderboards gives participants new learning experiences and motivates them to achieve higher rankings on learning leaderboards.

### Analysis of Leaderboard Cases Using the 3 Design Principles

**Figure 5:**
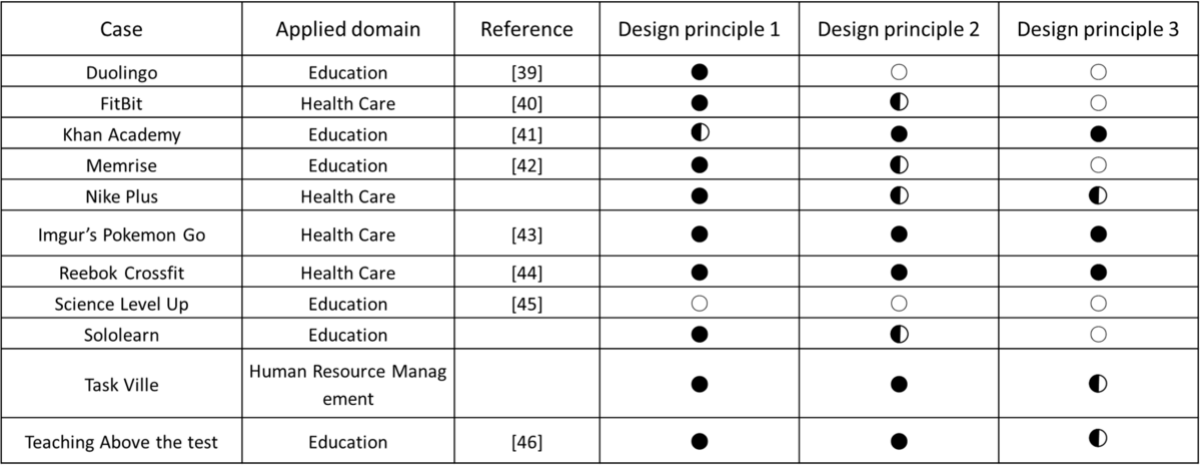
Collected leaderboard cases. ●: Completely applied; ◐: partially applied; and ○: not applied.

Figure 5 [38-45] shows the leaderboard cases collected for this research. We conducted a Google search to discover leaderboard cases. During our search for leaderboard cases, we aimed to find cases of educational gamification. During the leaderboard case collection stage, it was confirmed that the use of leaderboards in the sports field was frequent. Therefore, we expanded our search range to the sports field. The keywords used for our Google search were as follows: (1) leaderboard case(s), (2) gamified leaderboard case(s), (3) gamification leaderboard case(s), (4) leaderboard in sport, and (5) gamified leaderboard cases in sport. A total of 10 cases were collected and analyzed with the design principles developed in this study. The degree of application of each design principle is represented as ● (completely applied), ◐ (partially applied), and ○ (not applied) in Figure 5.

**Figure 6:**
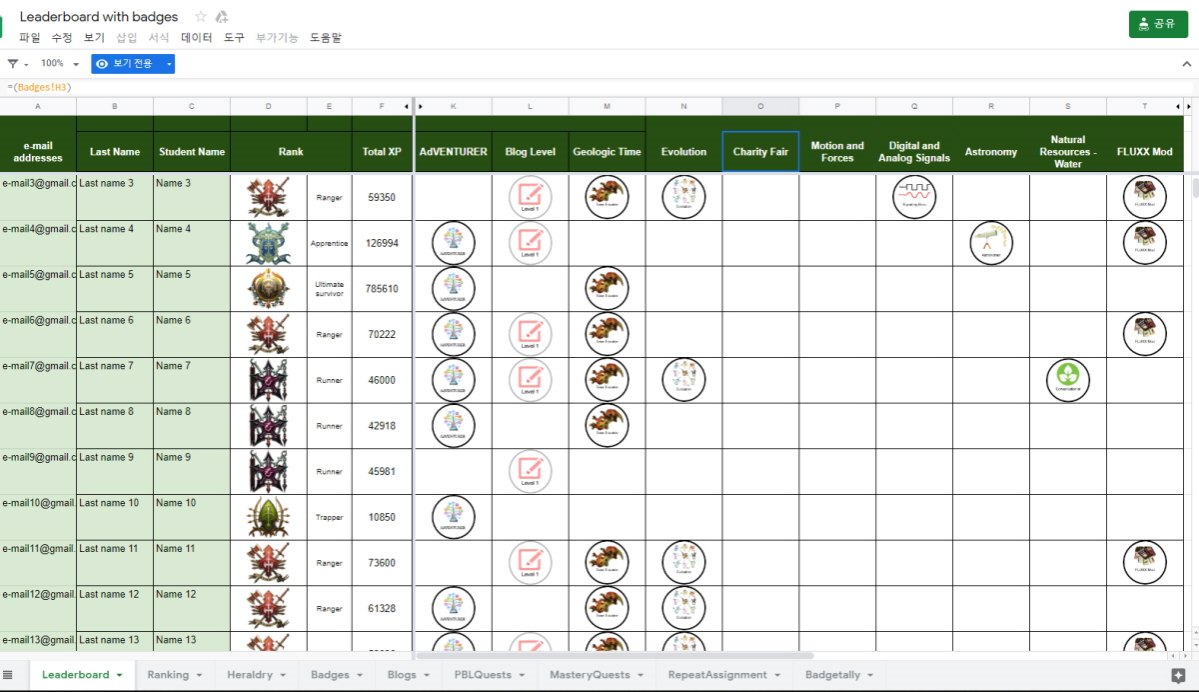
Leaderboard provided by Teaching Above the Test.

Duolingo, Khan Academy, Memrise, Science Level Up, and Sololearn are web-based learning platforms. Fitbit, NikePlus, Imgur’s Pokemon Go, and Reebok Crossfit’s “The Open” Challenge are cases related to health care. Pokemon Go is analyzed from the perspective of gamification because it is possible to monitor health through the game [46]. Teaching Above the Test is a leaderboard freely available through Google Sheets in gamified learning environments (Figure 6).

**Figure 7:**
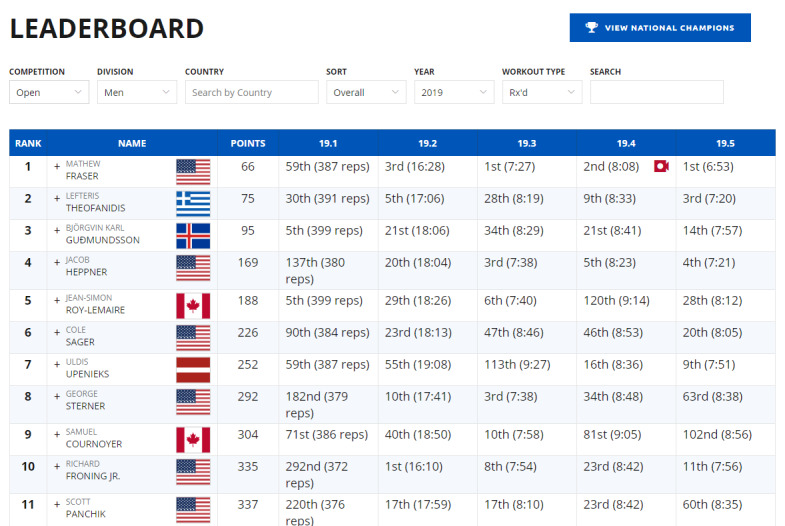
Leaderboard provided by Teaching Above the Test.

According to the analysis, the leaderboards on Reebok Crossfit’s “The Open,” Khan Academy, and Imgur’s Pokemon Go were found to be the most efficient. In contrast, Science Level Up’s leaderboard was the least efficient. Imgur’s Pokemon Go leaderboard lists all the badges obtainable in Pokemon Go and offers leaderboards according to region with relevant statistics. In addition, leaderboards are renewed in the form of monthly reports and published. The first leaderboard on the page is a macro leaderboard, and micro leaderboards are displayed by content underneath. Design principles 1, 2, and 3 have all been applied to this leaderboard. Figure 7 shows the leaderboard used in Reebok Crossfit’s “The Open” challenge. “The Open” is an event that has been ongoing since 2011. Anyone can participate and the event is held around the world simultaneously. The management designs a specific exercise as 1 set. Participants record 1 set during the period set by the management and the result is reflected on the leaderboard [47,48]. The main leaderboard webpage of “The Open” is a macro leaderboard. It shows the total points and ranks for each participant during the entire period. Users can set options to see micro leaderboards. This corresponds to principle 1 developed in this study. It is possible to see micro leaderboards by competition type (open, online qualifier, regionals, sectionals, games, team series, and liftoff), gender, and age group (divided into 5-year age groups from 10 years to 60 years), region, and year. This corresponds to principles 2 and 3 developed in this study. The strategies and physical competence of the players can be analyzed through the leaderboard. The Reebok Crossfit games webpage provides information on the types of exercises for each season. Based on the information, additional information about each player, such as his/her physical competence, characteristics, and strategies, can be obtained to compare with others who are ranked on the leaderboard. Thus, the exercises are designed to enable the leaderboard to be used from various perspectives and to provide the player with the ability to use the leaderboard as a means of improving performance.

**Figure 8:**
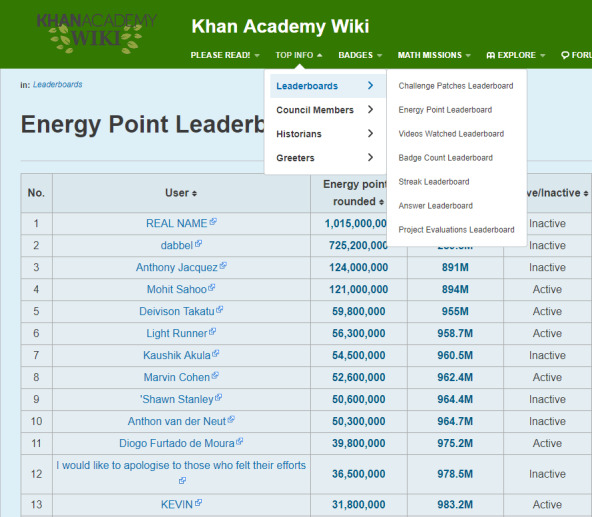
Leaderboard used in Reebok Crossfit’s “The Open” challenge.

Figure 8 shows the leaderboard of Khan Academy, which offers gamified programming learning content. The leaderboard provides micro leaderboards instead of a macro leaderboard. Leaderboards are provided for each of the elements identifiable as an indicator of learning activity in Khan Academy (challenge patches, energy points, video watching count, badge counts, streak stacks, answer counts, and project evaluation). This corresponds to principles 2 and 3 developed in this study. Web-based learning activities are presumed not to provide leaderboards reflecting precise gender and age groups. When users click on the identification of learners registered on the leaderboard, they can move to each player’s profile webpage to see detailed information. Khan Academy’s micro leaderboards reflect the number of badges acquired and social interactions such as the number of comments sent to other learners and programming training. This represents the principle 3 developed in this study. In contrast, Science Level Up was found to be the worst leaderboard. A macro leaderboard is not provided and micro boards are only offered by content. Additionally, only 1 micro leaderboard is provided for each content type, without differentiation by grade or demographic characteristics. None of the principles developed in this research are applied.

## Discussion

This study designed efficient leaderboards to motivate learners in gamified learning environments. Leaderboards are an efficient tool for competition and cooperation and can help learners set specific goals, boost learning motivation, and unleash affordances in the desired direction. However, as the gap between learners in terms of learning performance widens, learning motivation weakens. This study analyzed the negative influence of leaderboards on participants through a literature review and set the following 4 objectives for the design principles.

1. Objective 1: Minimize the sense of inadequacy.
2. Objective 2: Minimize learners’ experience of failure to minimize downward counterfactual thinking.
3. Objective 3: Maximize learners’ experience of success to induce upward counterfactual thinking.
4. Objective 4: Design leaderboards that measure learning performance to induce learners to obtain high scores and compete with each other frequently.

This study expects the following 3 leaderboard design principles to minimize the negative influence of leaderboards on participants and to improve leaderboard effectiveness. The design principles are as follows:

1. Design principle 1: Macro leaderboard and micro leaderboards should be designed and operated together.
2. Design principle 2: All the elements used to measure learners’ achievements in an educational environment should be incorporated into micro leaderboards.
3. Design principle 3: A “geeks leaderboard,” a type of micro leaderboard for activities other than learning, should be designed.

The negative influence of leaderboards should be controlled by gamification and teachers should promote affordances to guide learners in the right direction. Among game mechanics, leaderboards that encourage competition and cooperation based on social competence provide direct feedback to learners. Leaderboards should be designed and operated following appropriate design standards. Efficient leaderboard design principles are suggested in this study based on a literature review. In offline classrooms, a few instructors must control many learners and there are many items to manage. However, the introduction of gamification facilitates efficient classroom operation. There are cases of gamification that assist classroom operations such as class craft and class dojo. If leaderboards are designed and operated according to the leaderboard design principles suggested in this research, learning satisfaction and performance are expected to improve. When using leaderboards in web-based learning platforms as well as offline classrooms, this study recommends applying the principles developed in this study. Learners who experience leaderboards in other web-based environments perceive them as content rather than as a tool for competition or ranking [49]. Motivational affordances can be promoted through leaderboards unlike points or levels [50]. Individuals who have experienced leaderboards in other domains accept the competitive environment of the leaderboard in an educational context. We expect that this dynamic will motivate learners more naturally than other game mechanics. Thus, this study suggests that the leaderboard design principles developed here will enhance web-based gamified learning environments. Designers should refer to Reebok Crossfit’s “The Open” Challenge, Khan Academy, and Imgur’s Pokemon Go leaderboard.

This research has the following limitations. The suggested leaderboard design principles should be applied in education contexts and be verified for effectiveness. Basic leaderboard designs use leaderboards provided by the gamification system or formats shared on the internet. However, gamified leaderboards do not always produce positive effects. Therefore, the leaderboard design principles suggested in this research should be applied to leaderboard design and their effectiveness should be verified by learners. Glynn et al [51] developed the science motivation questionnaire 2 with reliability and validity guaranteed by statistical validation. The questionnaire is composed of intrinsic motivation, career motivation, self-determination, self-efficacy, and grade motivation factors. Future studies should design leaderboards following the leaderboard design principles developed in this study and use the survey tool to analyze the efficiency of the leaderboard design principles of this study in the education field. Furthermore, the leaderboards for use in other fields can be designed based on the results of this study, and the possibility of field expansion will be suggested through future studies.
